# Chemical-free recovery of crude protein from livestock manure digestate solid by thermal hydrolysis

**DOI:** 10.1186/s40643-021-00406-1

**Published:** 2021-07-12

**Authors:** Ken Tasaki

**Affiliations:** Tomorrow Water, 1225 N. Patt, Anaheim, CA 92801 USA

**Keywords:** Antioxidant, Cow manure, Protein hydrolysate, Thermal hydrolysis

## Abstract

Protein is becoming an increasingly important resource for a variety of commercial applications. Yet, a large volume of protein is being wasted. Notably, livestock manure solids have a significant content of protein which is not only underutilized, but prone to runoff and eventual breakdown to reactive nitrogen compounds, contributing to eutrophication. It would be desirable to remove protein before it causes environmental hazards and then convert it to value-added commercial applications. We have developed a novel thermal hydrolysis process (THP) to extract crude protein from livestock manure solid, or manure digestate solid (MDS) in particular, without the use of any chemical. We demonstrate the versatility of our new process to control the molecular weight (MW) distribution of the extracted protein hydrolysate (PH). The antioxidant activity of the crude protein hydrolysate (CPH) has been examined through Oxygen Radical Absorbance Capacity Assay. The results have shown that our CPH had its antioxidant capacity against the peroxyl radical similar to that of vitamin E and exhibited almost 7 times as strong inhibition against the hydroxyl radical as vitamin E. We also evaluated the nutritional value of our PH by analyzing its amino acid composition and the MW distribution through amino acid analysis, SDS-PAGE, and MALDI-TOF mass spectroscopy. The characterizations have revealed that the PH recovered from MDS had 2.5 times as much essential amino acids as soybean meal on dry matter basis, with the MW distribution ranging from over a 100 Da to 100 KDa. Finally, the protein powder was prepared from the extracted CPH solution and its composition was analyzed.

**Supplementary Information:**

The online version contains supplementary material available at 10.1186/s40643-021-00406-1.

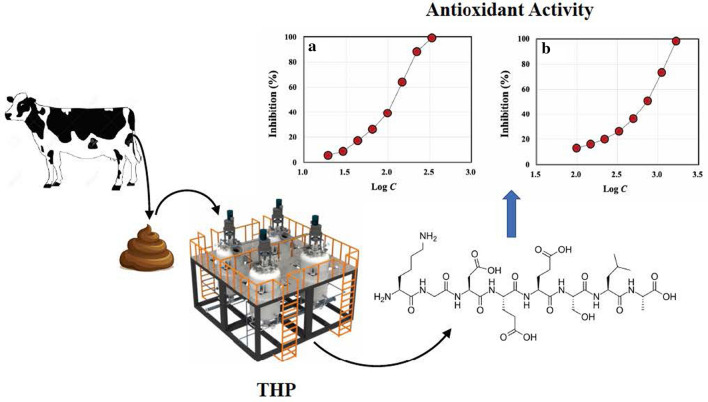

## Introduction

Livestock manure solids contain a considerable amount of crude protein, from 12 to 48 wt%, depending on the animal and the growth period, according to the report by Pacific Northwest National Laboratories (PNNL) (Chen et al. 2003). There is a substantially large volume of crude protein in livestock manure that could be potentially recovered (see Additional file 1: Table S1) (Livestock Wastes Subcommittee 1993; U.S. Department of Agriculture 2020). Currently, such crude protein is being wasted. Protein in manure tends to stay in the soil longer when manure is sprayed on croplands since it is not available to plants immediately as nutrient; hence, it can be subject to runoff to waterways or leaching to groundwater before the complete breakdown of protein, if not properly treated. Crude protein belongs to what is called biologically nondegradable organic nitrogen compounds (BNON) which are difficult to treat by conventional biological treatment processes (Kim et al. 2000). It would be beneficial if crude protein is recovered from manure before it causes environmental problems such as eutrophication and then the recovered crude protein is converted to value-added products from environmental and economical points of view.

Protein has been attracting increasing attention in biotechnology (Pasupuleti et al. 2010), nutrition science (Pasupuleti et al. 2010; McCalla et al 2010), and materials science (Dhillon 2016; Silva et al. 2014; Hu et al. 2012). In this report, we refer all the organic nitrogen compounds included in manure solids to as crude protein and designate true protein composed of amino acids (AAs) alone as protein. One important application of protein in manure solids, rarely explored, may be using its hydrolysate as antioxidants. Recently, a number of studies have shown that peptides with low molecular weight (MW) fractions have exhibited the antioxidant activity (Wu et al 2003; Adhikari et al 2019; Liu et al. 2016; Feng et al. 2017; Ye et al. 2018; Kim et al. 2019). Antioxidant peptides extracted from various food sources are attracting increasing interest because they provide a natural and safe alternative to many synthetic antioxidants (Hook et al. 2001). Compared to synthetic antioxidants, these peptides usually have relatively low MWs, stable structures and high activities, and are easily absorbed without hazardous immunoreactions (Qian et al. 2008). The range of MW for peptides having antioxidant activity has been reported to be < 3000 Da by one report (Kim et al. 2019) and 400–2000 Da by another (Liu et al. 2016). Mostly, enzymatic process was used for the extraction from various sources in the previous studies (Wu et al. 2003; Adhikari et al. 2019; Liu et al. 2016; Feng et al. 2017; Ye et al. 2018; Kim et al. 2019; Davalos et al. 2004; Sila and Bougatef 2016; Wang et al. 2018; Yang et al. 2018; Zou et al. 2016; Hook et al. 2001; Qian et al. 2008).

Vitamin E is a well-known antioxidant to alleviate the oxidative stress of animals. However, the price of vitamin E can be subject to the volatility and unexpected supply shortages. It would be desirable to have an alternative to vitamin E. The supply of livestock manure as the raw material is steady and uninterrupted, given the increasing global demand for meat.

A process of protein extraction from manure solid has been patented (Vanotti and Szogi 2019). The process uses a high concentration of alkali chemical ~ 1 M. The use of chemical requires not only separation of the chemical from protein, but recycle or disposal of the chemical as well. We have developed a chemical-free recovery process, using thermal hydrolysis process (THP). THP has been applied to the extraction of bioactive compounds from plants successfully (Nastića et al. 2018; Liang and Fan 2013). Very few reports have been published on the application of THP for protein recovery from manure solid in the literature.

For commercial application, making a powder out of the extracted protein is crucial, given the transportation of the final product to the market and the storage issue. Transportation of the protein solution can be costly, depending on the protein concentration.

We use the manure digestate solid (MDS) as the sample. It contains not only protein that was not digested by the animal body, but also cellular protein inside microbes such as methanogens that are rich in protein. To recover the protein from such microbes, the microbe cells need to be ruptured for recovery. Our objective in this study is to extract crude protein from MDS and hydrolyze it by THP and recover the hydrolysate by a shear wave-induced membrane ultrafiltration (SWIMU), using no chemical solvent, but only water. THP may be effective for extracting cellular protein inside microbes by breaking the cell membranes. Microbial cell disruption by THP has been reported (Baskar et al. 2019).

We examined the crude protein hydrolysate (CPH) recovered from MDS for the antioxidant activity, and the protein hydrolysate (PH) for the AA composition and the MW distribution. We also prepared a powder of the CPH solution by removing water and analyzed the composition of the powder. Our goal, however, is by no means to replace vitamin E, but rather to provide alternative antioxidants to livestock farmers.

## Materials and methods

### Materials

The MDS sample was collected from the solid separated by a screw separator from the digestate effluent of AD using manure of Jersey cattle as the feed on a dairy farm in California Central Valley. The MDS sample had a 50% water content after being left under the sun for several days. The MDS sample was used for THP without any pretreatment. Some of the samples were dried in an oven overnight to prepare dry samples for the composition analysis.

### Composition of materials

The composition of the MDS sample solid before and after THP was analyzed to determine the contents such as crude protein, hemicellulose, cellulose, lignin, phosphorous, potassium, and others. For the crude protein analysis, a combination of the flow injection analyzer by HACH (Hach Company, Ames, ISA) for the inorganic nitrogen (NO_3_ and NH_4_) and the combustion system for the total nitrogen (TN) was used. The crude protein content was estimated by subtracting the inorganic nitrogens (NH_4_, NO_3_, and NO_2_) from TN and multiplying the result by 6.25. The neutral detergent fiber (NDF), acid detergent (ADF), and acid detergent lignin (ADL) were measured by the Reflux method to analyze the lignocellulosic composition of the MDS sample (AAFCO 2017). The nutrients such as phosphorous and potassium, alkali metals, and other metals were analyzed by Inductively Coupled Plasma Atomic Emission Spectrometry (ICP-AES). Each lignocellulose component was calculated by the following: hemicellulose = NDF − ADF, cellulose = ADF − ADL, and lignin = ADL.

See Additional file 1: Composition of Materials, for the detailed instrumentation (AAFCO 2017).

### THP process

We have developed a two-heating step process for the extraction of crude protein from MDS by THP. The detailed description of the THP reaction vessel and the theoretical background are given in Additional file 1: Fig. S1. At the first heating step, we denature protein by heating MDS at the temperature *T*_1_ close to the denaturing temperature of the protein. At the second step at the temperature *T*_2_ (> *T*_1_) at which the solution becomes acidic, having a higher concentration of the hydronium ions, we perform hydrolysis of the extracted protein. Denaturing is essential to break down the protein to low-MW peptides by hydrolysis since the electrophile to break the peptide bonds cannot have sufficient access to the peptide bonds when the protein is folded. In addition, the water’s other unique characteristics, that is its density, dynamic viscosity, and surface tension all decrease significantly at high temperatures, promote the mass transfer of all the components in the solid matrices and increase the wettability of the protein molecule in the manure solid. We achieve the extraction/denaturing and hydrolysis of protein by the two-heating step process we have developed. As to the other nitrogen compounds, basically the same principle can be applied.

The temperature and the reaction time can control the extraction yield and the degree of hydrolysis of the protein. By adjusting the temperatures, we can control how much crude protein can be extracted from manure solid and how low the MW of the hydrolysate is. In determining the heating temperatures for the 1st and 2nd steps for our THP, considerations were given to the denaturing temperature of protein for the 1st step, while yielding as many low-MW fractions as possible for the 2nd step. As to the denaturing temperature, some proteins are known to denature at 50 °C (Olsson et al 2016), while many proteins denature irreversibly over 80 °C (Cavagnero et al. 1998; Pothekin et al. 2000; McAfee et al. 1996; Pfeil et al. 1997; Pappenberger et al. 1997). It is also possible that protein in the manure solid may well be denatured already. Since it is not known whether or not the protein in the manure solid is denatured, it was decided that the 1st heating temperature (*T*_1_) was fixed at 100 °C. As to the 2nd heating temperature (*T*_2_), 130 °C and 160 °C were chosen to examine the effect of *T*_2_ on the degree of protein hydrolysis. We chose two conditions for THP: *T*_1_ = 100 °C for 1 h followed by *T*_2_ = 130 °C for 1 h (to be referred to as THP I) and *T*_1_ = 100 °C for 1 h followed by *T*_2_ = 160 °C for 1 h (to be referred to as THP II). 60 g of MDS, 30 g of which was the dry matter, was mixed in 1 L of deionized water for THP without pretreatment. The CPHs under the two conditions, THP I and THP II, will be referred to as CPH I and CPH II, respectively. Likewise, PH I and PH II are the protein hydrolysate prepared under THP I and THP II, respectively.

### Protein filtration by ultrafiltration

SWIMU generates a vortex flow, by rotating the membrane disk, effectively preventing membrane fouling. The membrane fouling resistance can be reduced by as much as an order of magnitude, relative to a conventional cross-flow filtration (Kim et al. 2015). The reaction solution after the THP process was first filtered by a 90 mm mesh screen, followed by SWIMU with a 150 KDa membrane from which the permeate was recovered as the CPH sample which was used for the characterizations described in the following subsections. The detail of SWIMU is provided in Additional file 1: SWIMU (Kim et al. 2015). No separation of the PH from the rest of CPH was performed. Hence, the CPH sample was a mixture of PH and the non-protein nitrogen compounds.

### Sodium dodecyl sulfate polyacrylamide gel electrophoresis

The MW distribution of the samples was determined by sodium dodecyl sulfate polyacrylamide gel electrophoresis (SDS-PAGE). The samples were first diluted to a 2.5 mg/mL concentration in a sample buffer containing 2.3% sodium dodecyl sulfate (SDS), 10% glycerol, 50 mM dithiothreitol, and 63 mM tris buffer (pH = 6.8). Following the buffer addition, the samples were heated in a digital dry bath at 95 °C for 10 min. SDS slab gel electrophoresis was carried out using a 16.5% acrylamide peptide slab gel. The slab gel was stopped once the bromophenol blue front had migrated to the end of the slab gel. Following the slab gel completion, the gel was stained with Coomassie blue dye, destained in 10% acetic acid until a clear background was obtained and dried between cellophane sheets.

### MALDI-TOF mass spectroscopy

The matrix-assisted laser desorption ionization time-of-flight (MALDI-TOF) mass spectroscopy was performed to analyze the MW distribution of the samples using a Bruker ultraflextreme MALDI-TOF mass spectrometer (Bruker, Billerica, USA) with a 2 kHz SmartBeam laser for desorption and ionization of the samples. α-Cyano-4-hydroxycinnamic acid was used as the matrix. The original sample was diluted to 5 pmol for the measurements.

### Oxygen radical absorbance capacity against the peroxyl and hydroxyl radicals

For ORAC against the peroxyl radical, 2,2′-azobis (2-amidino-propane) dihydrochloride (AAPH) was used as the source for the peroxyl radical, which was generated as a result of the spontaneous decomposition of AAPH at 37 °C. Fluorescein was used as the chosen target protein, whose loss of fluorescence was an indication of the extent of damage from its reaction with the peroxyl radical. The protective effect of the antioxidants was measured by assessing the longer fluorescence time/intensity area under the curve of the sample compared to the blank, in which no antioxidant compounds were present. The concentration of the protein hydrolysate extracted from the MDS sample was 10%. The concentration of the protein hydrolysate was increased by circulating the concentrate from reverse osmosis filtration. Trolox, a water-soluble analog of vitamin E, was used as the calibration standard. On the other hand, the hydroxyl radical antioxidant capacity assay was performed by measuring the antioxidant capacity of the protein hydrolysate to protect fluorescein from damage by the hydroxyl radical which was generated from reactions between cobalt and hydrogen peroxide. The antioxidant capacity of the protein hydrolysate was measured by assessing the longer fluorescence time/intensity area under the curve of the protein hydrolysate compared to the blank, in which no sample was present. Trolox was used as the calibration standard.

All oxygen radical absorbance capacity (ORAC) assays were performed by Brunswick Laboratories (Southborough, MA).

### Amino acid analysis

The AA composition of the protein hydrolysate powder was analyzed by Hitachi L-8800 AA analyzer (Tokyo, Japan). Ion-exchange chromatography (IEC) was used to separate each AA followed by a post-column ninhydrin reaction detection system. Each sample was thoroughly hydrolyzed prior to IEC. Their standard hydrolysis procedure employed 6 N HCl for 24 h at 110 °C.

### Electron spin resonance spectroscopy analysis

THP may have generated radicals in the CPH samples. To determine the existence of radicals in the crude protein hydrolysate, electron spin resonance (ESR) spectroscopy was employed. The instrument was Bruker EMXPlus spectrometer (Bruker, Billerica, USA) operating at X-band frequency (~ 9.8 GHz) using a Bruker ER4119HS high sensitivity resonator (Bruker, Billerica, USA). Continuous-wave electron spin resonance spectra were recorded at room temperature. The modulation amplitude was 1.0 G, with the modulation frequency at 100 kHz, the conversion time of 5 ms, and the time constant of 2.56 ms. This analysis can help interpret the results of the antioxidant activity assay. One possibility for the antioxidant activity of the MDS hydrolysate, if any, is radical disproportionation in which two radicals are combined to form a non-radical compound. The result from this analysis can either eliminate the possibility or confirm the possibility.

### DNA analysis

Increasing antibiotic resistance genes in the environment is a serious public concern. Some antibiotic resistance genes have been found in livestock manure (Wichmann et al. 2014). Since THP can rupture microbial cells in the MDS sample, some genetic materials may stay in the reaction solution after THP. The analysis was performed for the CPH sample based on fluorescence quenching detection. Electropherogram was applied to quantify the genetic materials in the sample. The Maxwell DNA IQ kit (Promega, Madison, USA) was used for the extraction of the sample, while the QuantiFiler Trio kit (Thermo Fisher Scientific, Waltham, USA) was utilized for the quantification of DNA. The concentration limit by these instruments was 1 ppm. The detailed DNA analysis is given in Additional file 1: Composition of Materials. The concentration limit by these instruments was 1 ppm.

### Powder preparation

The powder was prepared by removing water from the CPH II aqueous solution by a combination of reverse osmosis (RO) and vacuum evaporation. The SWIMU permeate was further filtered by RO and circulated the concentrate from RO until the permeate stopped coming out of RO. The pressure applied to the RO membrane was 150 psi and the recovery rate was 80%. As a result, the concentration of the CPH II solution, or the SWIMU permeate, can be increased by fivefold by the RO process. The RO system was manufactured by Membrane Solution (Shanghai, China). The maximum flow rate was 150 gallons per day. The length and the diameter of the membrane element were 12″ and 2″, respectively. The concentrate from RO was vacuum evaporated under 700 mmHg at 40 °C to further remove water. The slurry in the flask after the vacuum evaporation was removed and dried in an oven overnight. The dried solid was ground by a pestle.

## Results and discussion

### Composition of materials

Table 1 summarizes the compositions of the original MDS sample and the leftover solid after THP on dry matter basis. The THP condition was THP II.

**Table 1:** Compositions of MDS samples before and the leftover solid after THP under THP II (wt%)

Solid sample	Crude protein	P	K	Hemicellulose	Cellulose	Lignin	Others^a^
Before THP	37.2	2.1	1.5	9.3	18.2	30.2	1.5
After THP^b^	14.8	3.5	0.1	12.7	24.9	41.5	2.5

Dry matter basis

^a^Alkali metals such as Na, Ca, and Mg

^b^The leftover solid after THP recovered by filtration by a screen with 90 mm mesh, dried in an oven overnight, and ground by a pestle for analysis

The crude protein content in the original MDS sample was higher than the value for cow manure solid previously reported by the PNNL group, 18.1 wt% (Chen et al. 2003). The manure solid composition can vary, depending not only on cow species, but also on the feed, the season, and the climate. In addition, our MDS likely contained cellular protein from microbes after anaerobic digestion. Still, the pure protein content was 30.7% based on AAA. The difference between the crude protein and the pure protein is due to the non-protein nitrogen compounds such as urea, humic acid, amino sugars, and others which are known to exist in manure solids (He et al. 2014; Khandelwal and Gaur 1980). The most significant compositional difference before and after THP in the solid is that more than half of the crude protein was dissolved from the original MDS sample under THP II. On the other hand, phosphorous mostly stayed in the leftover solid, while potassium dissolved in the solution after THP. As to the lignocellulosic compositions of MDS before THP, similar values have been reported (Tsapekos 2017; Zhong et al. 2016). After THP, the content of each lignocellulosic component actually increased in the leftover solid almost proportionally from that in the original MDS sample, as a result of the dissolution of crude protein. It suggests that they didn’t dissolve much into the solution during THP. A study on hot-water extraction from spruce wood has reported that delignification started at 170 °C, dissolving mostly hemicellulose from the wood (Krogell et al. 2013). Since our THP temperature was 160 °C, hemicellulose may not have dissolved much in the solution. Still, a direct comparison may be difficult, given that the lignocellulose in the MDS sample was partially digested during anaerobic digestion. The composition analysis of the protein powder, described later, will reveal more about the lignocellulose dissolution later. At least, any traceable amount of sugars such as glucose or xylan as a result of hemicellulose hydrolysis was not detected in the CPH sample.

Table 1 only lists the major components. Not included are other components such as alkali metals such as Ca, Na, and Mg in a few percentages and traces of other metals such as Fe, Mn, and Zn in a ppm range. The compositions of heavy metals such as Cd and As in the leftover solid were less than 10 ppm.

### Protein recovery yield

We focused on the pure protein for the recovery yield (RY), defined by the following equation:

1 $${\text{RY}} = 100 \times \frac{{\left[ {W_{{{\text{hydrolysate}}}} } \right]}}{{[W_{{{\text{protein}}}} ]}},$$

where [*W*_hydrolysate_] and [*W*_protein_] refer to the weight of the PH in the reaction solution after THP and the weight of the pure protein in the original sample prior to THP, respectively. Table 2 summarizes the recovery yields under THP I and THP II.

**Table 2:** Recovery yields under THP I and THP II

Condition	*W*_protein_, g^a^	*W*_hydrolysate_, g^a^	RY, %
THP I^b^	9.21	3.21	34.85
THP II^c^	9.21	5.55	60.26

^a^Dry matter basis

^b^*T*_1_ = 100 °C for 1 h and *T*_2_ = 130 °C for 1 h

^c^*T*_1_ = 100 °C for 1 h and *T*_2_ = 160 °C for 1 h

The numbers listed in Table 2 were determined by AAA. *W*_protein_ was the weight of protein in the MDS sample of 60 g used for THP, of which 30 g was the dry matter. *W*_hydrolysate_ was the weight of PH in 1 L of reaction solution after THP. The experimental error of RY was within 3% which was estimated over triplicate experiments. The higher second heating temperature significantly increased the recovery yield. We attribute this to the increased thermal energy at the 2nd heating step that facilitated the protein extraction. From Table 1, the dissolved crude protein was about 6.73 g out of 30 g of the dry MDS under THP II. With the dissolved protein of 5.55 g, the dissolved non-protein was about 1.18 g. One of the non-protein nitrogen compounds, urea decomposes to NH_3_ and biurea at 130 °C (Tischer et al. 2019). The decomposition temperature of biurea is 230–260 °C (Russell and Strachan 1978). Thermogravimetric data have shown humic acid started decomposing around 200 °C (Antunes et al. 2007), while thermal decomposition studies on amino sugars are scarce. They were all likely dissolved in the solution after THP. Vanotti et al. did not include the recovery yield in their patent (Vanotti and Szogi 2019).

### MW distributions

**Fig. 1:**
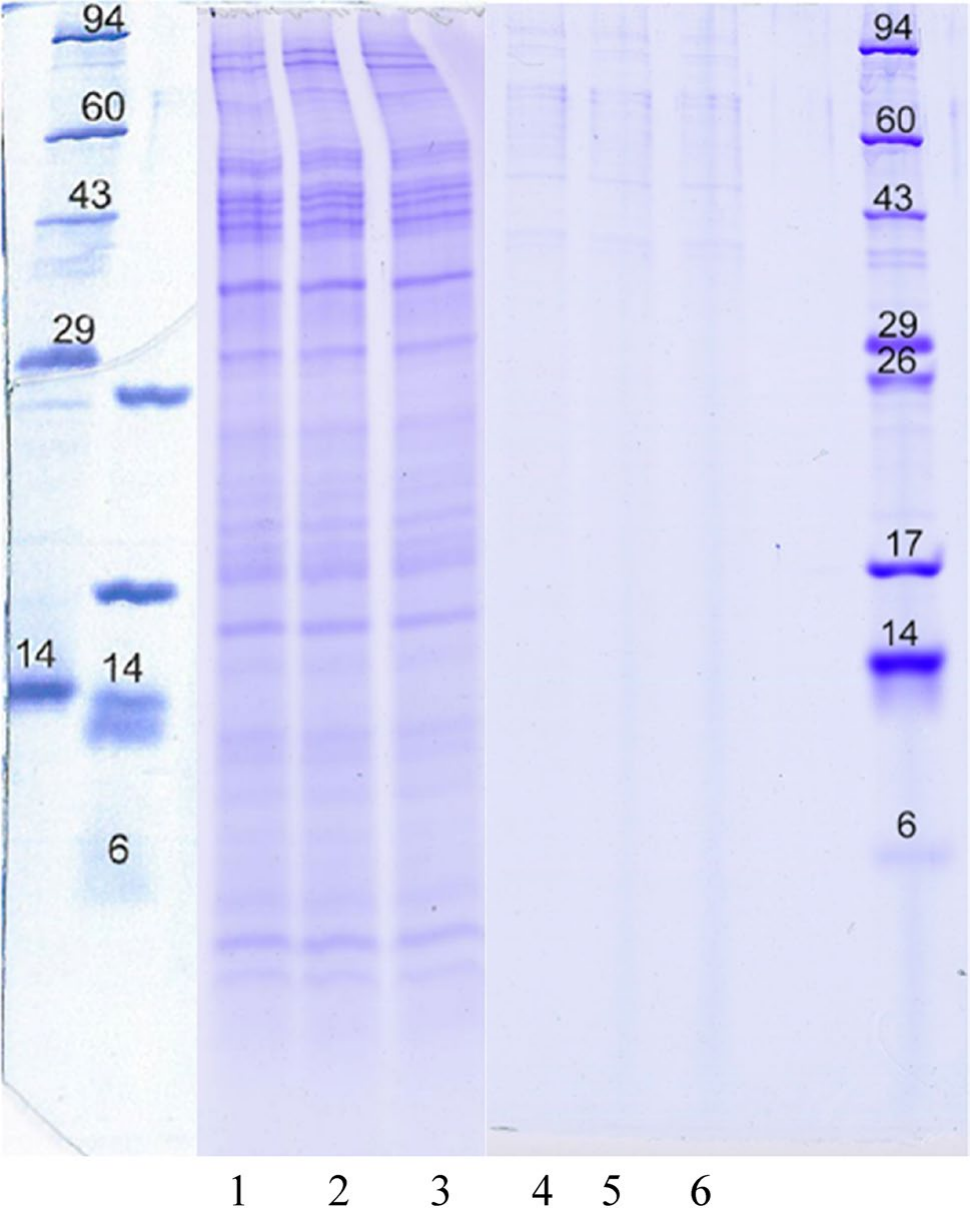
SDS-PAGE images of the PHs extracted from MDS: 1–3—PH I and 4–6—PH II. The measurements were performed triplicate. The numbers on both sides are the MW markers in KDa for which the following proteins were used as the standards: phosphorylase A (94,000), catalase (60,000), actin (43,000), carbonic anhydrase (29,000), myoglobin (17,000), lysozyme (14,000), aprotonin (6500), insulin chain b (3496)

Figure 1 displays the SDS-PAGE band images for the PHs prepared under THP I and THP II. CPH I exhibited the continuous bands with some dark distinctive bands around 30 KDa, 40 KDa, 50 KDa, and a few lines up to 95 KDa, demonstrating a wide range of high MW fractions. On the other hand, CPH II showed very few lines, indicating almost no fractions within the range analyzed. The bands shown in Fig. 1 are primarily due to protein, not non-protein nitrogen compounds, the MW of which are mostly below 1 KDa.

**Fig. 2:**
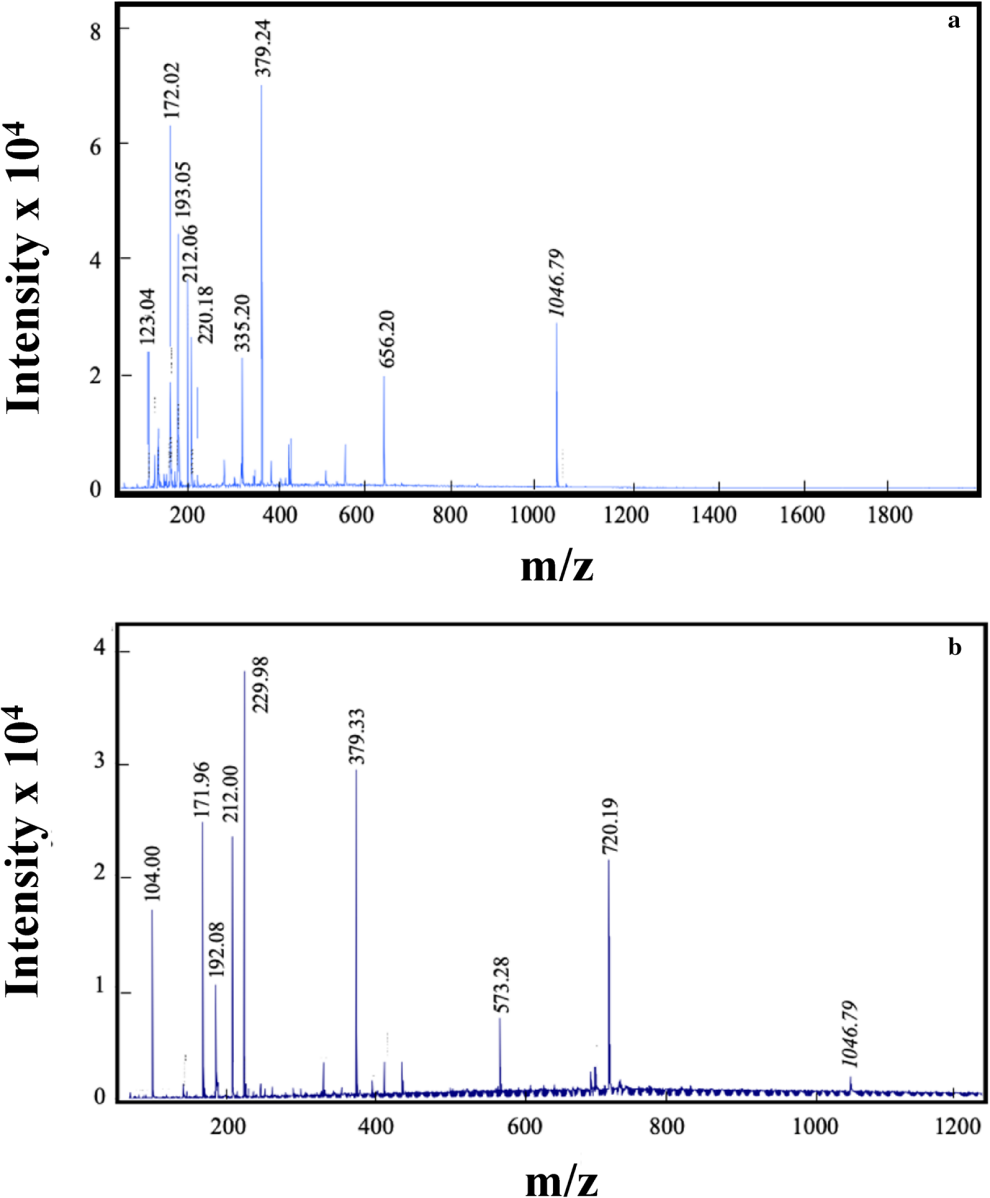
MALDI-TOF-mass spectra of **a** CPH I and **b** CPH II. A signal for a peptide with a known MW is included as a reference at 1046.79 m/z

Figure 2a, b exhibits the MALDI-TOF mass spectra for CPH I and CPH II, respectively. The reference peptide, shown at 1046.79 m/z, was added to the sample prior to MALDI-TOF-mass spectroscopy measurements for comparison between the two samples. Though several peaks were seen in both charts, based on the peak height of the reference peptide, the concentration of low-MW fractions in CPH II was much higher than that in CPH I below 1000 m/z within which most peaks appear. Peptides in this region were low-MW peptides such as oligopeptides or free AAs. The concentration of the hydrolysates in Fig. 2b can be calculated from the peak positions and the intensities of each signal relative to the reference. It was about 2.9 g/L which is close to the value of *W*_hydrolysate_ in Table 2. We believe that the difference in the MW distribution between THP I and THP II is due to *T*_2_ for THP II (> *T*_2_ for THP I) which provided a higher hydronium concentration, resulting in more rigorous hydrolysis, breaking protein into smaller peptides. pH is 5.9 and 5.7 at 130 °C and 160 °C, respectively (Plaza and Turner 2017). Based on the difference in pH, THP II had about 60% more hydronium concentration than THP I.

Based on the results from MALDI-TOF mass spectroscopy and SDS-PAGE, we conclude that CPH II had much more low-MW fractions than CPH I which had its fractions more distributed over higher MW ranges, as is demonstrated by SDS-PAGE. Accordingly, we will focus on CPH II in the following characterizations since earlier studies reported low-MW peptides exhibited antioxidant activities.

### ORAC against the peroxyl and hydroxyl radicals

**Fig. 3:**
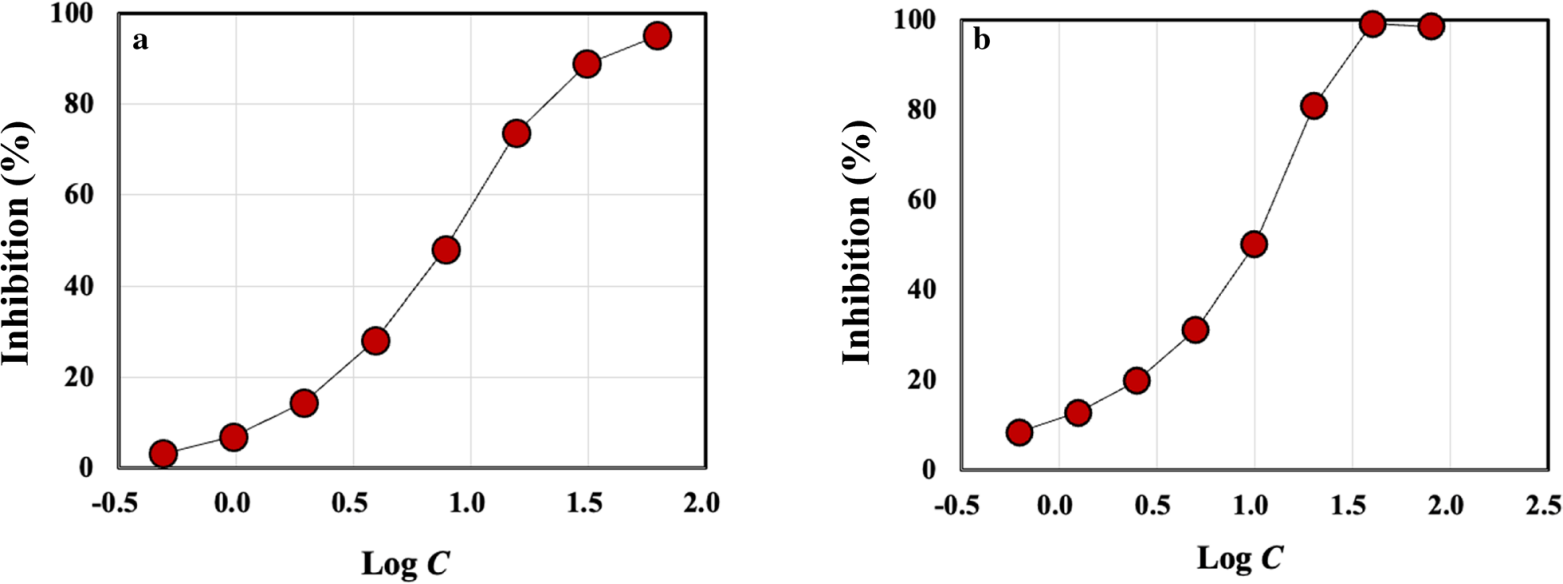
The inhibitions of the peroxyl radical by **a** Trolox and **b** CPH II in percentage as a function of logarithm of the CPH II concentration, *C* in mg/L

Figure 3a, b plots the inhibition of the peroxyl radical attack against fluorescein protein by Trolox and CPH II, respectively, as a function of the logarithm of the sample concentration, *C*. The two curves exhibit similar profiles to one another, though Trolox reached 100% inhibition at a somewhat lower concentration than CPH II. Still, the value of IC_50_ for CPH II, 7.67 mg/L, was very close to that of Trolox, 8.08 mg/L, demonstrating that the antioxidant activity of CPH II was as strong as Trolox against the peroxyl radicals. IC_50_ refers to the concentration of the sample at which the inhibition is 50%. The experimental error for IC_50_ was within 1 mg/L which was estimated over triplicate experiments.

**Fig. 4:**
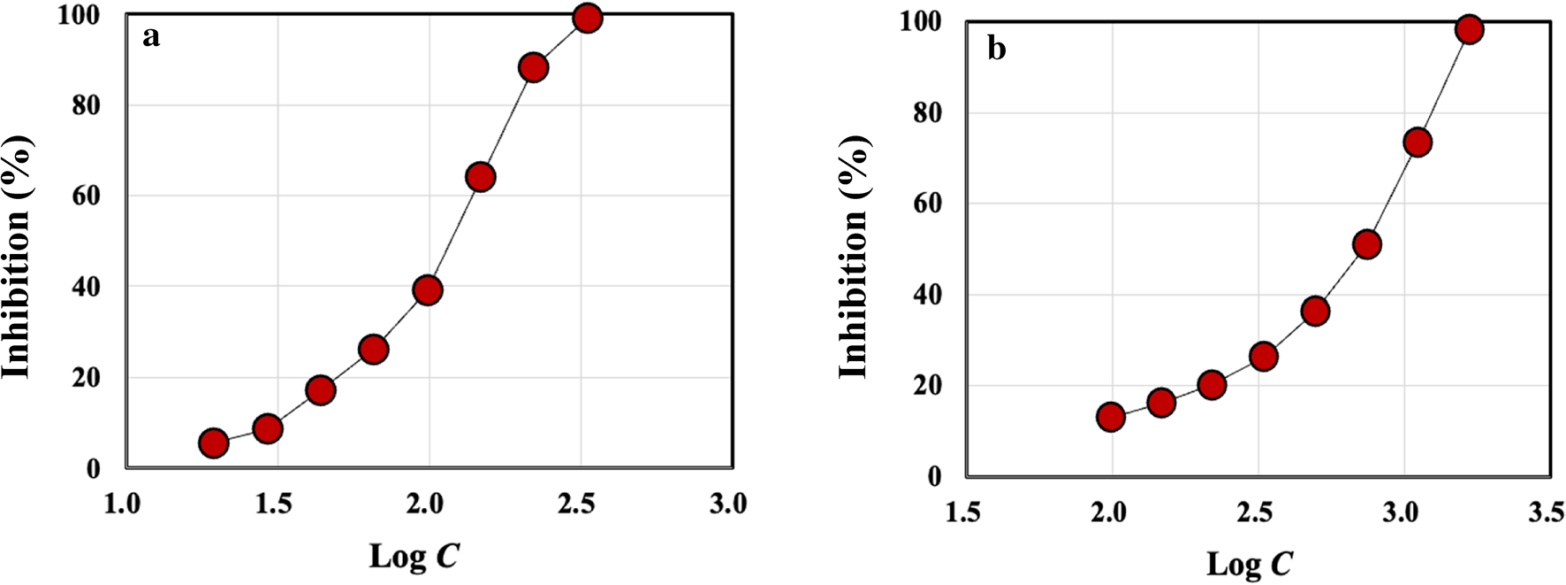
The inhibitions of the hydroxyl radical by **a** Trolox and **b** CPH II in percentage as a function of logarithm of the CPH II concentration, *C* in mg/L

Figure 4a, b shows the inhibition of the hydroxyl radical attack against fluorescein protein by Trolox and CPH II, respectively, as a function of the logarithm of *C*. Here, we observed a significant difference between the two samples: the inhibition by CPH II reached 100% at a much lower concentration than Trolox did, with IC_50_ of 107.6 mg/L, less than 1/7 of that of Trolox, 741 mg/L. The result suggests that the antioxidant activity of CPH II was more than 7 times as strong as Trolox. The observation that CPH II exhibited antioxidant activities is consistent with the previous studies on peptides (Wu et al. 2003; Adhikari et al. 2019; Liu et al. 2016; Feng et al. 2017; Ye et al. 2018; Kim et al. 2019; Davalos et al. 2004; Sila and Bougatef 2016; Wang et al. 2018; Yang et al. 2018; Zou et al. 2016; Hook et al. 2001; Qian et al. 2008). A theoretical study on the antioxidant activity of peptides has been published (Leung et al. 2018).

It should be noted that the sample CPH II includes not only PH, but also the other nitrogen compounds such as humic acid, amino sugars, and biurea extracted from the original MDS sample. Both humic acid and amino sugars have hydroxyl groups which can involve in the mechanisms associated with inhibition of radicals through the electron or the hydrogen atom transfer. In fact, some studies have reported the antioxidant activities of humic acid and some amino sugars (Kanmaz et al. 2016; Kitts et al. 2012; Banaszkiewicz 2011). There are also a number of studies showing strong antioxidant activities of peptides, as was mentioned earlier. These non-protein nitrogen compounds were about 17% of the total dissolved crude protein, as was described above. Since we did not remove these nitrogen compounds from the CPH II sample, their contributions to the inhibition of the radicals cannot be ignored. Our data only demonstrate that the extracted compounds from the MDS sample and recovered by UF with 150 KDa inhibited both peroxyl and hydroxyl radicals, to the extent that the ability to inhibit the former radical is comparable to that of Trolox and the ability in inhibiting the latter is 10 times stronger than Trolox.

### AA composition

**Fig. 5:**
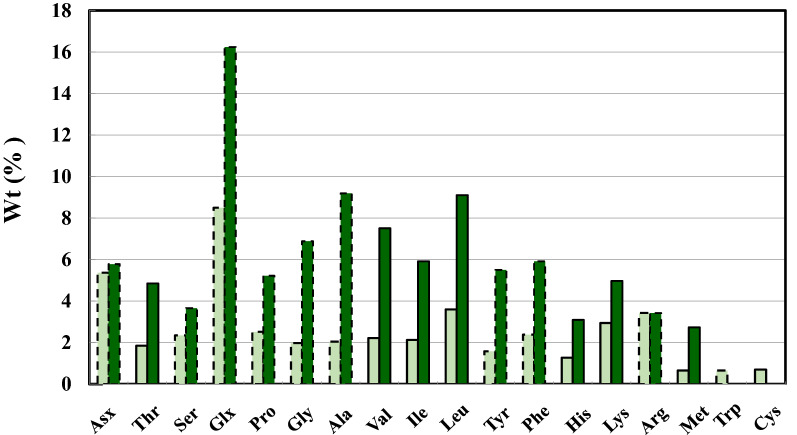
AA compositions of PH II, shown by the dark green bars. The ordinate represents the wt% on dry matter basis, while the abscissa refers to the AA residues. The light green bars represent the AA composition of soybean meal. The bars with the broken line refer to unessential AAs, while those with the solid line are the essential AAs for hog and poultry. *Asx* asparagine and aspartate, *Glx* glutamine and glutamic acid

Figure 5 compares the AA composition of PH II (PH under THP II) and that of soybean (the white bars) on dry matter basis (Banaszkiewicz 2011). The experimental error was within 1–2% in terms of wt% of each AA determined. The cysteine content of PH II was low due to the oxidation of the thiol during THP. According to Fig. 5, CPH II had 2.5 times as much essential AAs as those of soybean meal on dry matter basis. Further, it had 2.3 times as much nitrogen source as that of soybean meal on dry matter basis. The low-MW distribution of PH II may help its digestion by animals. The use of PHs has been growing in animal nutrition as an important nutrient (McCalla et al. 2010). Still, feed tests such as digestibility tests need to be conducted for further confirmation for PH II to be an effective feed additive. The difference in the content of the essential AAs between PH II and soybean meal is mainly because the protein content in PH II was 75.17% on dry matter basis, as shown below, while the protein content in soybean meal is 47.5%.

### CPH powder

Table 3 summarizes the composition of the powder prepared from the CPH II sample.

**Table 3:** Composition of CPH II powder (%)

Crude protein	P	K	Hemicellulose	Cellulose	Lignin	Cd	Pb	As	Others^a^
90.96	1.97	4.41	0.32	0.04	0.01	0.1^b^	10.3^b^	1.5^b^	1.99

Based on dry matter

^a^Alkali metals such as Na and Mg, sugars such as glucose and fructose

^b^In ppm

The components listed in Table 3 were the total dissolved solids (TDS) recovered from the permeate of SWIMU and rejected by the RO membrane. The powder showed a high content of crude protein, of which 75.17% was protein based on AAA and 15.78% was the other nitrogen organic compounds by subtracting the amount of the protein from the amount of the crude protein. The potassium content was relatively high, given more potassium being dissolved than phosphorous by THP. Very low components of the lignocellulosic components were found in the powder. Accordingly, it is very unlikely that the antioxidant activities are due to the lignocellulosic components. The others include alkali metals such as sodium and magnesium. The concentrations of heavy metals such as arsenic, cadmium, and lead were less than 10 ppm.

It is known that manure can contain prion (Tamgüney et al. 2009). It has been reported, however, that the conversion of a normal prion to an aberrant scrapie isoform triggering transmissible spongiform encephalopathies involves the formation of a new intermolecular disulfide bond (Welker et al. 2001). Our AAA indicates that no cystine residue was found in PH II. Cystine is the AA having the disulfide bond. In fact, THP oxidizes the disulfide bond, releasing the H_2_S gas. The destruction of the disulfide bonds in the misfolded prion results in a loss of the prion’s tertiary structure, hence removing its ability to transmit the misfolded shape onto a normal form of prion.

### DNA analysis

Prior to DNA analysis for CPH II, a number of known DNA samples were tested for calibration of the instrument, showing the expected cycle threshold (CT) values. For CPH II, however, no fluorescence was observed above the threshold over PCR amplification; hence, no CT was obtained to determine the concentration of genetic materials in our sample. The observation demonstrated that the binding of a fluorescent detector molecule to DNA molecules did not occur in our sample and therefore, no genetic materials were detected by the analysis, at least those higher than 1 ppm. Accordingly, no chart is presented. This result strongly suggests that no genetic materials higher than 1 ppm were extracted by THP from the MDS sample. A number of studies have reported that the application of THP significantly reduced the abundance of antibiotic resistant genes and mobile genetic elements in sewage sludge (Tong et al. 2017; Pei et al. 2016; Ma et al. 2011). It has been suggested that during THP, the high temperature and pressure sterilized the sludge floc and destroyed cell walls, which made the degradable components readily released (Tong et al. 2017; Pei et al. 2016; Ma et al. 2011; Donoso-Bravo et al. 2011). It has been also reported that DNA was susceptible to thermal hydrolytic destruction (Tong et al. 2017).

### ESR spectroscopy

The ESR spectroscopy chart for CPH II exhibited only noise signals. Additional file 1: Fig. S2 shows the ESR spectra. The sample was measured at various power levels, starting with the lowest power level, in an attempt to locate an EPR signal without causing saturation effects. No signal characteristic to a single electron spin, or radical, was observed in the range studied. The result indicates that radicals were not present in the CPH II sample, at least no more than 1 ppm. However, radicals were possibly produced during THP, but the radicals may have been short-lived through disproportionation. ESR spectroscopy was performed a day after the sample preparation. On the other hand, the ORAC assay was performed several weeks after the sample preparation. Based on this observation, it seems unlikely that CPH II inhibited either the peroxyl or the hydroxyl radicals through radical disproportionation. On the other hand, likely other mechanisms such as electron transfer, hydrogen atom transfer, and proton loss may be at work (Pham-Huy et al. 2008). However, discussion on the mechanism is beyond the scope of this work. We will report the radical scavenging mechanism by CPH II in a separate study.

## Conclusion

We have successfully extracted protein from MDS and hydrolyzed it by our two-step SWE process under two conditions and recovered it by the SWIMU filtration system. This process can be used to remove protein from manure before reaching the environment and convert it to value-added products. Once protein can be removed from manure solids, there will be less BNON left in the solids, hence easier for biological waste treatment.

We also have demonstrated that the protein hydrolysate characteristics can be controlled by adjusting the SWE condition. For example, we found that PH I treated by SWE at *T*_2_ = 130 °C had high MW fractions, > a few thousand Da, while PH II treated at *T*_2_ = 160 °C had lower MW fractions, < 2000 Da, based on SDS-PAGE and MALDI-TOF mass spectroscopy. The ORAC assays demonstrated that PH II had strong antioxidant activities against both peroxyl and hydroxyl radicals, achieving a nearly 100% inhibition of the radicals. Especially, the results suggested that the antioxidant activity of PH II was almost 7 times as strong as Trolox. ESR spectroscopy detected no radical for PH II, removing a possibility of the reduction of the peroxyl or the hydroxyl radicals by radical disproportionation. Still, the antioxidant assays conducted in this study should be regarded as preliminary. More extensive study is warranted on the antioxidant activity of protein hydrolysates extracted by SWE. It is also of importance to note that the antioxidant activity tests reported here were in vitro tests. In vivo and toxicology tests will be required before petitioning to FDA for approval as animal dietary supplements, among other tests. As to the nutritional aspect of our protein hydrolysate, we have found that the AA composition of PH II had 2.5 times as much essential AAs as those of soybean meal and 2.3 times as much nitrogen source as that of soybean meal on dry matter basis. The nutritional value of the protein hydrolysate will require validation through animal tests.

### Supplementary Information

**Additional file 1.** Supplementary material. 1. Global protein generation by livestock animals. **Table S1.** Estimated global volume of protein generated by livestock animals. 2. THP reactor vessel. **Fig. S1.** The cross section of the reaction vessel for THP. 3. Two-step THP. 4. Supplement to materials and methods. 4.1 Composition of materials. 4.2 SWIMU filtration. 5. Supplement to results. 5.1 ESR spectroscopy. Fig. S2. ESR chart of PH II. 6. Other supplemental materials.

## Data Availability

The Additional file is available.

## Abbreviations

AA: Amino acid
AAA: Amino acid analysis
AAPH: 2,2′-Azobis (2-amidino-propane) dihydrochloride
AD: Anaerobic digestion
BNON: Biologically nondegradable organic nitrogen compounds
CPH: Crude protein hydrolysate
CT: Cycle threshold
ESR: Electron spin resonance
MDS: Manure digestate solid
MW: Molecular weight
ORAC: Oxygen radical absorbance capacity
PH: Protein hydrolysis
PNNL: Pacific Northwest National Laboratories
RO: Reverse osmosis
RY: Recovery yield
SS: Suspended solids
SWIMU: Shear wave-induced membrane ultrafiltration
TDS: Total dissolved solids
THP: Thermal hydrolysis process
THP I: THP condition with *T*_1_ = 100 °C for 1 h and *T*_2_ = 130 °C for 1 h
THP II: THP condition with *T*_1_ = 100 °C for 1 h and *T*_2_ = 160 °C for 1 h
