# Supplementary material for: Chemical-free recovery of crude protein from livestock manure digestate solid by thermal hydrolysis
Source: Bioresour Bioprocess. 2021 Jul 12;8(1):60. doi: 10.1186/s40643-021-00406-1 (PMC10991932; doi:10.1186/s40643-021-00406-1)
Supplement: Supplementary file 1 — Additional file 1. Supplementary material. 1. Global protein generation by livestock animals. Table S1. Estimated global volume of protein generated by livestock animals. 2. THP reactor vessel. Fig. S1. The cross section of the reaction vessel for THP. 3. Two-step THP. 4. Supplement to materials and methods. 4.1 Composition of materials. 4.2 SWIMU filtration. 5. Supplement to results. 5.1 ESR spectroscopy. Fig. S2. ESR chart of PH II. 6. Other supplemental materials. [file 40643_2021_406_MOESM1_ESM.docx]

**Additional file 1**

Chemical-free recovery of crude protein from livestock manure digestate solid by thermal hydrolysis

Ken Tasaki

Tomorrow Water

1225 N. Pat St.

Anaheim, CA 92801

USA

*Correspondence should be addressed to Ken Tasaki E-mail: kt@bkt21.com

Table of Content

1. **Global Protein Production by Livestock Animals** S3

**Table S1**. Estimated Global Volume of Protein Generated by Livestock Animals S3

1. **THP Reactor Vessel** S4

**Fig. S1**. The cross section of the reaction vessel for THP S5

1. **Two-Step THP** S5
2. **Supplement to Materials and Methods** S6

**4.1 Composition of Materials** S8

**4.2 SWIMU Filtration** S9

1. **Supplement to Results** S11

5.1 **ESR Spectroscopy**  S11

**Fig. S2.** ESR chart for PH II. No signal for radical, but only noises are shown in a wide range of magnetic field. S11

1. **Other Additional Materials** S12
2. **Global Protein Generation by Livestock Animals**

**Table S1**. Estimated Global Volume of Protein Generated by Livestock Animals

|  | Protein^a^  % | Manure^b^  kg/day/head | Solid^c^  % | Dry Manure^d^ kg/day/head | Protein^e^ kg/y/head | Head Counts^f^  million | Protein^g^  MMT/y |
| --- | --- | --- | --- | --- | --- | --- | --- |
| Dairy | 18.1 | 25.8 | 12.7 | 3.3 | 216.7 | 144 | 31.2 |
| Cattle | 12.1 | 22.5 | 11.6 | 2.6 | 138.6 | 987 | 136.8 |
| Hog | 25.1 | 4.7 | 9.2 | 0.4 | 36.4 | 654 | 23.7 |
| Poultry | 39.8 | 0.1 | 25.2 | 0.0 | 2.1 | 68,566 | 146.8 |
| Total |  |  |  |  |  |  | 338.7 |

^a^The protein content in manure on dry matter basis (Chen et al., 2003).

^b^The weight of manure discharged a day per head of animal (Midwest Plan Service, 1993).

^c^The solid content in manure (Midwest Plan Service, 1993).

^d^The weight of dry manure discharged a day per head of animal (Midwest Plan Service, 1993).

^e^The weight of protein discharged a day per head of animal.

^f^The global head counts of each livestock animal (USDA, 2020).

^g^The annual weight of protein discharged by livestock animal globally in million.

1. **THP Reactor Vessel**

Fig. S1 illustrates a bench-scale THP reaction vessel used for this study. The gas inlet and outlet were closed during THP since no gas was used during THP. The reactor vessel for THP had the volume capacity of 2 liters, made of stainless steel, surrounded by a heat jacket capable of heating up to 300 °C. Two temperature sensors were used to monitor the temperatures inside and outside the vessel, the difference of which was used to determine the heating rate which was controlled by a temperature controller. After the sample was placed inside the reactor vessel, the vessel top cover was sealed by a stainless-steel vessel lid by hydraulic pressure to withstand high pressures which were the saturated water vapor pressures at given temperatures. A stirrer was attached to the bottom of the vessel lid for stirring the sample inside the vessel during the THP reaction. After the THP reaction was completed, water was circulated through a cooling coil to cool down the reaction solution, while the internal pressure was released through the gas outlet. Once the internal pressure was equalized to the atmospheric pressure and the reaction solution was cooled down to ambient temperature, the top lid was opened, and the reaction solution was discharged from the outlet at the bottom of the reaction vessel. The system was designed for a multi-step heating. For example, the sample can be heated at a given temperature for a given time after which the subsequent heating at another temperature can be started automatically, controlled by a control panel.

***b***

***c***

***d***

***e***

***g***

***h***

***h***

***i***

***j***

***k***

***e***

***f***

***a***

**Fig. S1**. The cross section of the reaction vessel for THP: *a*—a stirrer; *b*— a vessel lid; *c*—a gas inlet; *d*—a gas outlet; *e*—a temperature sensor; *f*—a reaction vessel; *g*—a ceramic wall; *h*—a heating coil; *i*—a reaction solution outlet; *j*—a cooling coil.

1. **Two-Step THP**

The unique nature of water has been well documented (Plaza and Turner 2017). For example, water becomes a good solvent for hydrophobic molecules, but a poor solvent for hydrophilic compounds at high temperatures. This is due to the dielectric constant of water decreasing significantly at high temperatures: it drops by about half in going from 20 °C (ε = 78.5) to 160 °C (ε = 41.2) (Plaza and Turner 2017). It follows that hydrogen bonding maintaining the protein tertiary structure becomes less pronounced at high temperatures, facilitating the denaturing of protein in a manure solid. Another benefit of a decrease in the dielectric constant is that water becomes less polar at high temperatures, starting to hydrate hydrophobic parts of the protein, weakening the hydrophobic interactions not only within the protein molecule, further destabilizing the protein structure, but also between the protein molecule and the surrounding other components such as cellulose, hemicellulose, and lignin in the manure solid. For example, at ambient temperature, protein molecules may be tightly embedded in the solid matrices through van der Waals forces or hydrogen bonds. At high temperatures, kinetically activated water molecules may go into voids or pores in the swollen solid matrices, sneaking in between the protein molecules and the other molecules, breaking the hydrogen bonds, weakening the van der Waals interactions, and eventually solubilizing the protein molecules. The water’s other unique characteristics, that its density, dynamic viscosity, and surface tension all decrease significantly at high temperatures, promote the mass transfer of all the components in the solid matrices and increase the wettability of the protein molecule in the manure solid. For example, the density, dynamic viscosity, and surface tension of water decrease significantly from 0.997, 0.891, and 72.0 at 25 °C to 0.915, 0.182, and 47.5 at 160 °C, respectively (Plaza and Turner 2017). Hence, water acts as an excellent extraction solvent for protein from manure solid at high temperatures. The effect of water on the other nitrogen compounds in the crude protein should be similar. The extraction and denaturing of protein from manure solid is the first heating step in our THP.

The next step is to hydrolyze the protein by heating up further, now that the protein is denatured. The water dissociation constant, *K_w_* = [H_3_O^+^][OH^-^]/[H_2_O]^2^, can increase as much as two orders of magnitude in going from 20 °C to 160 °C ((Plaza and Turner 2017). In fact, pH decreases to 5.7 at 160 °C (Plaza and Turner 2017). Hence, H_3_O^+^ can act as an oxidizing agent, breaking the peptide bonds, hydrolyzing the protein into peptides, oligopeptides, or amino acids, depending on the temperature and the reaction time. This is our two-step heating process for extraction/denaturing and hydrolysis of crude protein from manure solids.

1. **Supplement to Materials and Methods**
   1. **Composition of Materials**

A combination of the flow injection analyzer by HACH (Hach Company, Ames, ISA) for the inorganic nitrogen (NO_3_ and NH_4_) and the combustion system for the total nitrogen (TN) were used to determine crude protein. Nitrate was determined by reduction to nitrite via a copperized cadmium column. The nitrite was then determined by diazotizing with sulfanilamide followed by coupling with N-(1-naphthyl) ethylenediamine dihydrochloride. The absorbance of the product was measured at 520 nm. Ammoniacal nitrogen was determined by heating with salicylate and hypochlorite in an alkaline phosphate buffer. The presence of EDTA prevented precipitation of calcium and magnesium. Sodium nitroprusside was added to enhance sensitivity. The absorbance of the reaction product was measured at 660 nm and was directly proportional to the original ammonia concentration. This method had a detection limit of approximately 0.10 ppm. The combustion system was equipped with an induction furnace coupled with a thermal conductivity detector system and an IR detector system. The analytical method was based on the oxidation of the sample by “flash combustion” which converted all organic and inorganic substances into combustion gases (N_2_, NO_x_, CO_2_, and H_2_O). The method had a detection limit of approximately 0.02% for nitrogen. The neutral detergent fiber (NDF), acid detergent (ADF), and acid detergent lignin (ADL) were also measured by the Reflux method to analyze the lignocellulosic composition of the MDS sample (AAFCO, 2017). The detection limit for NDF, ADF, and ADL was approximately 0.5 %. The nutrients such as phosphorous and potassium, alkali metals, and other metals were analyzed by Inductively Coupled Plasma Atomic Emission Spectrometry (ICP-AES). The method detection limit was either 0.1 mg/L or 0.1 meq/L.   For the composition analysis of the leftover solid after THP, the solid was recovered by filtration by a screen with 90 mm mesh, dried in an oven overnight, and ground by a pestle for analysis. The crude protein content was estimated by subtracting the inorganic nitrogens (NH_4_, NO_3_, and NO_2_) from TN and multiplying the result by 6.25. Each lignocellulose component was calculated by the following: hemicellulose =NDF – ADF, cellulose =ADF – ADL, and lignin=ADL.

- 1. **SWIMU Filtration**

The reaction solutions after the THP treatment contained a large volume of suspended solids (SS). Hence, the color of the solutions was opaque. The SS was primarily due to fibers in the MDS. Visible solids were first removed from the reaction solution by a 90 mm mesh screen, the filtrate of which was further filtered by SWIMU with a 150 KDa membrane to remove all other undissolved components. The protein hydrolysate was recovered from the permeate of the filtration. For SWIMU, we applied is a vortex flow generating ultrafiltration system called FMX^®^ which effectively removed all TSS from the reaction solution after THP (Kim et al., 2015). After the FMX^®^ filtration, the permeate solution was clear, implying very few SS left. Membrane fouling is a serious issue especially when organic matters such as protein are present, giving rise to frequent membrane washing or replacement. Since FMX^®^  reduces membrane fouling by generating the vortex flow, the filtration continued for extended period of time without membrane washing. The pressure applied to the membrane was 3 bar. The permeate from the FMX^®^ filtration was collected for the characterizations. With a 150 KDa membrane used for the FMX^®^ filtration, the permeate was expected to include not only protein hydrolysates, but minerals, soluble phosphate, soluble potassium. It may have also included sugars as a result of hemicellulose hydrolysis, and possibly some genetic materials after microbial cell membrane rapture by THP.

**5. Supplement to Results**

**5.1 ESR Spectroscopy**


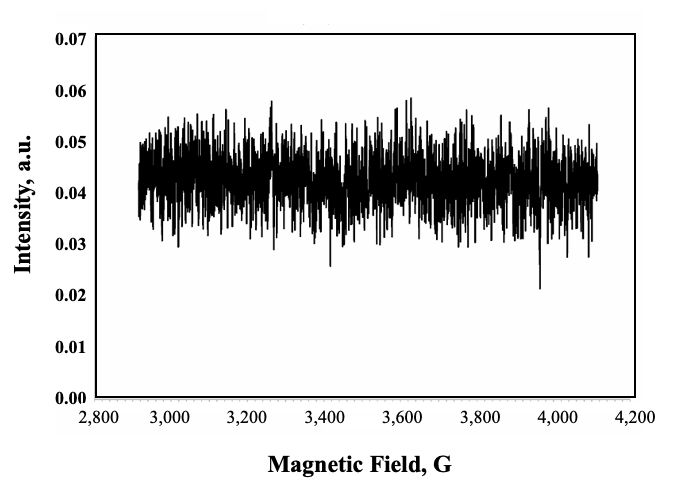


**Fig. S2**. ESR chart of PH II. No signal for radical, but only noises are shown in a wide range of magnetic field.

**6. Other Additional Materials**

Antibiotics are often found in livestock manure. We did not perform any analysis on antibiotics on either PH I or II sample. Some studies have shown that antibiotics such as sulfonamides, macrolides, lincosamides, and tetracycline were mostly removed by THP at 160 °C from wastewater at municipal wastewater treatment facilities.^58,59^ One advantage of THP is that the process under high temperatures and high pressures can sterilize manure/digestate, killing pathogens such as *E coli* or *O157:H7*. As to Bovine Spongiform Encephalopathy (BSE), it is possible to remove it by hydrolysis which breaks down the misfolded protein called prion to individual AAs or peptides, thus prion losing its tertiary and quaternary structural characteristics which are essential for the cause of BSE. Still, this is merely a speculation and will need to be validated by analysis. As to the heavy metals, if any, most of them in manure have been reported to stay in the solid residue after THP.^60^
